# Aminoglycoside riboswitch control of the expression of integron associated aminoglycoside resistance adenyltransferases

**DOI:** 10.1080/21505594.2020.1836910

**Published:** 2020-10-24

**Authors:** Jun Zhang, Getong Liu, Xuhui Zhang, Yaowen Chang, Shasha Wang, Weizhi He, Wenxia Sun, Dongrong Chen, Alastair I.H. Murchie

**Affiliations:** a Key Laboratory of Medical Epigenetics and Metabolism, Fudan University Pudong Medical Center, Institutes of Biomedical Sciences, Fudan University, Shanghai, PR China; b Key Laboratory of Metabolism and Molecular Medicine, Ministry of Education, School of Basic Medical Sciences, Fudan University, Shanghai, PR China

**Keywords:** Aminoglycoside, Riboswitch, Adenyltransferase, Antibiotic Resistance

## Abstract

The proliferation of antibiotic resistance has its origins in horizontal gene transfer. The class 1 integrons mediate gene transfer by assimilating antibiotic-resistance genes through site-specific recombination. For the class 1 integrons the first assimilated gene normally encodes an aminoglycoside antibiotic resistance protein which is either an aminoglycoside acetyltransferase (AAC), nucleotidyltransferase – (ANT), or adenyl transferase (AAD). An aminoglycoside-sensing riboswitch RNA in the leader RNA of AAC/AAD that controls the expression of aminoglycoside resistance genes has been previously described. Here we explore the relationship between the recombinant products of integron recombination and a series of candidate riboswitch RNAs in the 5ʹ UTR of *aad* (aminoglycoside adenyltransferases) genes. The RNA sequences from the 5ʹ UTR of the *aad* genes from pathogenic strains that are the products of site-specific DNA recombination by class 1 integrons were investigated. Reporter assays, MicroScale Thermophoresis (MST) and covariance analysis revealed that a functional aminoglycoside-sensing riboswitch was selected at the DNA level through integron-mediated site-specific recombination. This study explains the close association between integron recombination and the aminoglycoside-sensing riboswitch RNA.

## Introduction

The aminoglycoside antibiotics are among the earliest classes of antibiotics to be discovered and are used clinically to treat severe bacterial infections. They target the A site in the decoding region of the 30S ribosomal subunit and cause mistranslation of mRNA in the translocation process of bacterial translation [1–3]. Aminoglycoside resistance is usually caused by enzymatic modification of the drug, methylation of rRNA target site or overexpression of efflux pump proteins [4]. Enzymatic acetylation, adenylation, and phosphorylation of the aminoglycosides can prevent them from binding to their target site, the ribosomal A site, leading to resistance. The enzymes that catalyze acetylation or adenylation of aminoglycoside are aminoglycoside acetyltransferases (AAC) or adenyl transferases (AAD) (also known as aminoglycoside nucleotidyl transferases (ANT)) [5].

Historically, integrons were first discovered through the spread of antibiotic resistance. As mobile genetic elements, integrons accumulate antibiotic-resistance genes through site-specific recombination. The wide-spread application of antibiotics in the clinic created an environment where integron-mediated site-specific recombination on resistance plasmids led to the rapid acquisition of resistance genes and the proliferation of resistance plasmids (reviewed in [6]). Five integron classes (classes 1–5) have been identified based on amino acid similarities in the Int1 integrase protein sequences [7]. Class 1 integrons comprise a site-specific recombinase encoding integrase gene (*int1 1*), recombination site (*attI 1*) for integrase binding and the insertion of resistance gene cassettes (that contain an attC recombination site and a resistance gene), and two appropriately orientated (divergent) promoters, one promoter (P_int_) drives the integrase gene and the other (P_c_) transcribes the cassette genes [8,9]. Site-specific recombination mediated by integrons on plasmids carrying the AAC or AAD contributes to the wide spread of drug resistance driven by over use of aminoglycosides [6,10].

Asymmetric cassette recombination between the bottom strands of the *attC* site and the *attI1* site ensures that cassettes are inserted in the correct orientation for expression from the integron Pc promoter [11,12]. Expression from the promoter is limited to the first cassettes of the array, and downstream cassettes may be regarded as a low-cost reservoir which may be accessed by recombinational shuffling [13]. In the type 1 integrons, start codons vary from the normal AUG, to UUG and GUG [14,15] and SD sequences comprise 3 or more bases of the sequence UAAGGAGGUGA which are complementary to the 3ʹ-end of the small subunit 16S rRNA (e.g. AGGU) [14].

The expression of resistance genes enables bacteria to survive in an environment rich in antibiotics, but in the absence of antibiotics their expression has significant metabolic cost. To reduce this fitness burden and ensure a proportional response, the expression of resistance genes is regulated [16,17]. Regulatory mechanisms of transcriptional and translational attenuation by structured RNAs have been described since the 1980s [18,19], and for resistance to ribosomal antibiotics, mechanisms of translational [20–22] and transcriptional attenuation have been described [23] (reviewed by [24] and [25]). The riboswitches are structured non-coding regulatory RNAs commonly located in the 5ʹ untranslated regions (UTRs) of messenger RNAs (mRNAs) that bind small-molecule metabolites or cofactors to regulate transcription and translation of related genes and control the metabolic pathway of the organism (reviewed in [26,27]). The aminoglycoside antibiotics are the products of complex biosynthetic pathways [28–30]. As natural product antibiotics, the aminoglycosides are secondary metabolites and as such are not merely inhibitors of bacterial growth, but may also have regulatory roles as effector molecules for a range of biological activities, and may be considered to be modulators [30,31]. They have been shown to induce T-box transcriptional riboswitch activity [32], sub-inhibitory doses can induce biofilm formation [33], they also have organism-specific effects on the bacterial SOS response [34]. An aminoglycoside-sensing riboswitch which is present in the leader RNA of the resistance genes that encode AAC and AAD was discovered in 2013 [35]. The riboswitch RNA can specifically bind certain aminoglycosides to induce a structural transition in the RNA which leads to the induction of the *acc/aad* gene expression. Reporter assays showed that specific aminoglycosides can induce expression of a reporter gene mediated by the riboswitch RNA. Direct binding between the aminoglycosides and the riboswitch RNA could be measured by Surface Plasmon Resonance (SPR). Chemical probing experiments and gel electrophoretic mobility assays further proved that aminoglycoside binding to the riboswitch RNA induced a structural transition in the RNA. A specific cross-link between the aminoglycoside sisomicin and the RNA was detected. Overall, the aminoglycosides that induced reporter gene expression displayed higher affinity for the leader RNA by SPR, its binding also caused a structural transition in the RNA. The induction of reporter gene expression through riboswitch binding to aminoglycosides can also take place in the presence of resistant ribosomes. For the published *aac* riboswitch of *P. fluorescens*, a model was put forward based on results from chemical probing data and mutational analysis; the RNA has tandem ribosome-binding sites (SD1 and SD2) and in the absence of the aminoglycosides, SD2 (GGAG) is sequestered by an anti-SD sequence (CUUC) which prevents ribosomal access; binding of aminoglycosides causes a structural change and migration of the anti-SD to base pair with SD1 (GGAG) thus making SD2 available for translation of the resistance gene (Figure 4(a)). Subsequent covariance analysis of *aac* riboswitch RNAs from 13 resistant bacterial pathogen strains generated covariant RNA structures and confirmed the general features of the model for the structure transition [36].

At the RNA level, the aminoglycoside-sensing riboswitch induces *aac/aad* gene expression leading to aminoglycoside resistance. At the DNA level, the initial acquisition of the aminoglycoside resistance is mediated by class 1 integrons on plasmids carrying the *aac/aad* gene. The DNA sequence corresponding to the aminoglycoside-sensing riboswitch RNA therefore overlaps with the conserved *attI1* site of the class 1 integrons. The relationship between the integron DNA sequence and the coincident riboswitch at the 5ʹ UTR of *aac* (aminoglycoside acetyltransferases) gene was investigated; sequence analysis of clinical aminoglycoside resistant strains and biochemical evidence showed that site-specific recombination mediated by the integron preserves the function of the aminoglycoside-sensing riboswitch of *aac* genes [36]. In this paper, we focus on the relationship between integron and riboswitch RNA in the 5ʹ UTR of the *aad* (aminoglycoside adenyltransferases) genes. Here we have analyzed RNA sequences from the 5ʹ UTR of *aad* genes from pathogenic strains that are the end product of site-specific DNA recombination by class 1 integrons. These new sequences were analyzed by reporter assays and MicroScale Thermophoresis (MST). Covariance analysis data revealed an aminoglycoside-sensing riboswitch mechanism that was preserved at the DNA level by the selection of integron-mediated site-specific recombinants. This study reveals a correlation between the class 1 integron and an aminoglycoside-sensing RNA for *aad* genes and also further confirms the original discovery of aminoglycoside-sensing riboswitch.

## Materials and methods

### Chemicals and reagents

Superscript III reverse transcriptase, ribonucleotide triphosphate (rNTP), RNase inhibitor, DNase I, fluorescein-5-thiosemicarbazide (FTSC) were purchased from Invitrogen. T7 RNA polymerase was purified in-house. Aminoglycoside antibiotics and oligonucleotide primers were purchased from Sangon Biotech. All other reagents were purchased from Sigma-Aldrich unless otherwise stated.

### Bioinformatics analysis

The published 75nt minimal aminoglycoside riboswitch RNA sequence was used to blast search the NCBI database (Nucleotide collection (nr/nt)) for more homologous sequences using default settings with an upper limit of 5*10^3^ sequences [35]. Duplicate sequences were removed and subsequently, the top 131 homologous *aad*B related sequences were input into CMfinder to produce local multiple sequence alignments of the structurally conserved motifs, that contained the motifs previously established by chemical probing [35,37]. The structure was manually revised based on the published structure and used to construct a covariance model incorporating new searches that were conducted in the RefSeq database version 85 [38] using infernal [39]. RNA structures were drawn using R2R [40] and Adobe Illustrator.

### Reporter construction and reporter assays

The new riboswitch candidate sequences were cloned into the reporter plasmid pGEX-leaderRNA-*aac/aad*-lacZα as described before [35]. Agar diffusion assay on plates [41] and miller assays in solution [42] were performed as described previously.

### MicroScale thermophoresis

The riboswitch RNAs were prepared by in vitro transcription using T7 RNA polymerase. Purified RNA was labeled with FTSC as previously described [35]. The FTSC labeling RNA and the antibiotics were both prepared in 10 mM HEPES (pH 7.5), 100 mM KCl, 0.1 mM MgCl_2_. The RNA was annealed by heating at 95°C for 2 minutes followed by slowly cooling down to room temperature. MST experiments were conducted in triplicate on a Monolith NT.115 system (NanoTemper Technologies) and data analysis was performed by MO.Affinity analysis software [36,43].

## Results

### *The association of the aminoglycoside-sensing riboswitch RNA of the* aad *genes with the integron*

**Figure 1.**
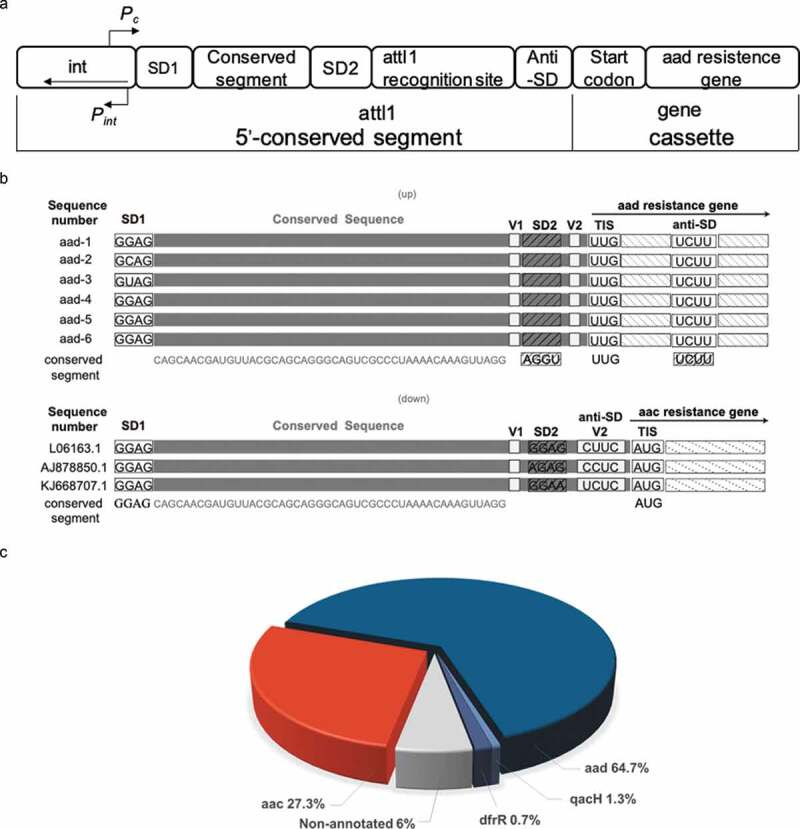
The sequence analysis of *aad* riboswitch RNAs

Seventy-five nucleotides of the aminoglycoside riboswitch *aac/aad* RNA was confirmed experimentally as the minimal functional unit [35]. The DNA sequence of the 75 nt riboswitch RNA partially overlaps with the integrase binding site and the 7 bp 5ʹ attI1 insertion site of class 1 integrons (Figure 1(a)) [9,44]. This DNA sequence is located between the (divergent) integrase and the resistance proteins aminoglycoside acetyl or adenyltransferases. From Blast searches 150 sequences similar to the 75 nt riboswitch RNA were selected; the majority of the sequences were next to annotated genes of known function comprising 97 aminoglycoside adenyltransferases (*aad)* and 41 acetyltransferases (*aac*) as shown in Figure 1(c), which has been further updated by manual annotation [35,45]. The analysis of the RNA sequences upstream of the *aac* genes showed that integron-driven site-specific recombination preserves the functional riboswitch RNAs that can induce expression of *aac* genes in the presence of aminoglycosides [36]. The main disparity between the riboswitch RNAs of the *aad* and *aac* genes is at the 3ʹ end and reflects the different origins of the resistance cassettes (Figure 1(b), Figure 4(b,c), supplementary Table 1). Here we have focused on the analysis of the regulatory mechanism of the riboswitch RNA upstream of the *aad* genes. Similar riboswitch RNA sequences upstream of *aad* genes can be found in the integrons of numerous antibiotic-resistant pathogens such as *Pseudomonas aeruginosa, Salmonella enterica, Klebsiella pneumonia* (supplementary Table 1). The riboswitch RNAs are organized such that they retain the 5ʹ portion of the attI1 site that is highly conserved and includes the first ribosome-binding site (SD1). The 3ʹend of the RNA, adjacent to the *aad* coding sequence shows greater sequence variation (Figure 1(b)) into the coding sequence of the resistance gene.

(a) The conserved 5′ conserved segment of class 1 integrons. The strong promoter (Pc) [9] transcribes the inserted gene cassette (i.e., *aad* resistance gene). While the divergent promoter P_int_ transcribes the integrase (*int*) gene. Single gene cassettes are inserted by int-mediated recombination between int recognition sites. The *int* expression is linked to the SOS response that can only be induced by non-aminoglycoside antibiotics in *E. coli*. (b) The leader *aad* riboswitch RNAs from different pathogens. The RNA is located between the divergent *int* and *aad* genes at the conserved 5′ end of the integron. (c) The blast search results of the *aac/aad* riboswitch sequence from *P. fluorescens*. A total of the 141 of the top 150 BLAST sequences were annotated to identify the neighboring genes. The majority of neighboring resistance genes encoded AAD (n = 97) and AAC (n = 41). One non-aminoglycoside resistance gene dihydrofolate reductase (*dhfR*, 1) and a nonfunctional multidrug exporter (*qacH*, 2) were also noted.

### *The aminoglycoside riboswitch of* aad *RNA from various pathogenic strains mediates reporter gene expression*

**Figure 2.**
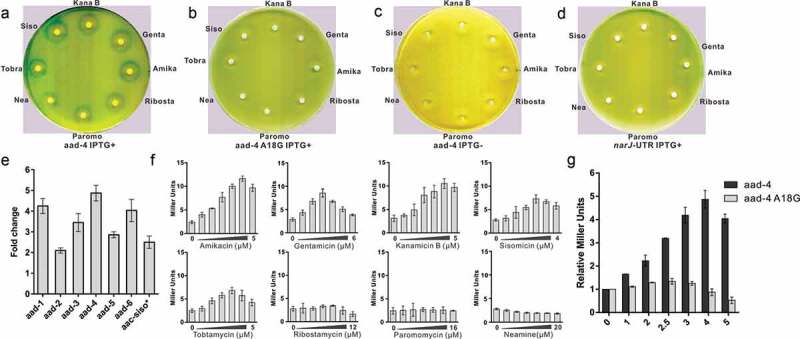
Reporter gene expression controlled by *aad* riboswitches

The reporter plasmid pGEX-leaderRNA-*aac/aad*-lacZα was previously used for functional analysis of the riboswitch RNA of *aac/aad in vivo*. In the plasmid, the sequence of the 75nt *aac* riboswitch RNA in *P. fluorescens* was placed under the control of the IPTG-inducible tac promoter (P_tac_) and upstream of a β-galactosidase (β-gal) reporter gene. Certain aminoglycosides induced reporter gene expression through the riboswitch RNA as measured by Miller assays in solution and agar diffusion assays on plates. Specific point mutations in the riboswitch RNA of *P. fluorescens* or unrelated UTR RNAs of *cat-86, ermCL* or *ghoS* genes were shown to cause a loss of reporter gene induction [35,36]. Here, a number of *aad* riboswitch RNA sequences were identified. To compare the function of these *aad* riboswitch RNAs with the published riboswitch RNA, six riboswitch RNA sequences were chosen (Figure 1(b), supplementary Table 2). The DNA sequence corresponding to each of the RNA sequences and an unrelated RNA (*narJ*) [46] as controls were cloned into the plasmid pGEX-leaderRNA-*aac/aad*-lacZα. The reporter plasmid was transformed into the *E. coli* strain JM109 and β-gal activity was assessed in the presence of aminoglycoside antibiotics by agar diffusion assays and Miller assays. In agar diffusion assays, on addition of IPTG, RNA is synthesized, and in the presence of the 4–6 deoxystreptamine aminoglycosides sisomicin, gentamicin, kanamycin B (KanB), amikacin or tobramycin, zones of reporter gene expression were observed for all six riboswitch RNAs, in contrast, only faint levels of reporter gene expression could be detected for neamine and ribostamycin, or paromomycin (Figure 2(a) for the sequence *aad*-4 and Supplementary Figure S2 for the other sequences *aad*1-3 and *aad* 5–6). Miller assays showed clear 4–6 deoxystreptamine aminoglycoside dependent induction of reporter gene expression, for all six sequences and only minimal levels of expression for neamine, ribostamycin and paromomycin (Figure 2(f) for *aad*-4 and supplementary Figure S1 for the other tested sequences). The results from the agar diffusion assays and the Miller assays are clearly consistent. In the absence of IPTG no reporter gene expression was observed for *aad*-4 with the antibiotics (Figure 1(c)). For the control construct for the *narJ* RNA no induction of reporter gene expression was observed in the presence of aminoglycosides (Figure 2(d)) in contrast to the wild-type RNA. These results suggest that the induction of reporter gene expression requires both a specific aminoglycoside and the presence of the riboswitch RNA of the *aad* gene. Therefore, the six *aad* derived RNAs studied here retain their riboswitch function and activate downstream gene expression in the presence of inducing aminoglycosides. The riboswitch sequences analyzed here came from clinical strains and are the products of integron-mediated site-specific recombination which has selected functional riboswitches that activate adenyl transferase resistance genes in an aminoglycoside rich environment.

**(a)** Agar diffusion assay of *E.coli* transformed with *aad*-4 reporter construct plasmid with aminoglycoside antibiotics in the presence of IPTG. **(b)** Agar diffusion assay of the reporter construct plasmid with aminoglycoside antibiotics for the *aad*-4 A18G point mutation in the presence of IPTG. **(c)** IPTG control agar diffusion assay of the aad-4 reporter construct plasmid with aminoglycoside antibiotics in the absence of IPTG. **(d)** Control RNA agar diffusion assay of the *NarJ* reporter construct plasmid with aminoglycoside antibiotics in the presence of IPTG. **(e)** The highest induction fold change for the *aad* riboswitch constructs (*aad* 1–6) in the presence of amikacin. The *aac*-siso* refers to the highest induction fold of the *P. fluorescens* riboswitch RNA under sisomicin [35]. **(f)** The β-gal activity (Miller units) of the reporter gene with *aad*-4 riboswitch sequence upon titration of aminoglycoside antibiotics. **(g)** Comparison of the fold change in β-gal activity of the wild-type aad-4 sequence with the inactive A18G point mutation on titration of amikacin to 5 µM. NB for all β-gal activity measurements, error bars are standard deviations of at least three independent experiments.

Initially, of the aminoglycosides, sisomicin gave the highest induction of reporter gene expression in the *aac* riboswitch RNA [35], and this analysis has been expanded to show that specific *aac* riboswitch sequences can distinguish between different aminoglycosides for optimal levels of reporter gene induction [36]. Here for each of the *aad*-1-6 riboswitch RNAs, the highest induction of β-gal was observed with amikacin. In the presence of amikacin, *aad*-1, *aad*-4, and *aad*-6 mediate the induction of reporter gene expression by up to 4–5 fold, which is higher than that in the originally published riboswitch sequence, and comparable to the most recently analyzed sequences [36], both *aad*-2 and *aad*-5 were also capable of inducing reporter gene expression to similar levels to the published *aac* riboswitch [35] (Figure 2(e)). Therefore, these variant RNA sequences are capable of enhancing reporter gene expression through interacting with amikacin. The distinct specificities of the aminoglycosides for different *aad* and *aac* [35,36] riboswitch RNAs could only be due to specific interactions between the riboswitch RNA and the aminoglycosides because the ribosome or other cellular components in the system are the same and the only difference is the riboswitch RNA sequence.

### *Binding of aminoglycosides to the* aad *riboswitch RNAs*

**Figure 3.**
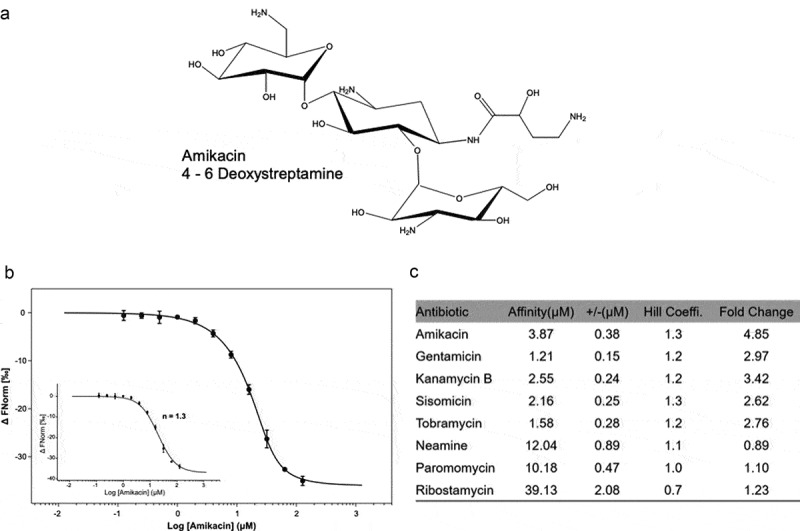
The binding affinity of aad-4 riboswitch RNA with aminoglycoside antibiotics

The aminoglycosides bind directly to the riboswitch RNA of *Pseudomonas fluorescens*. The binding has been measured by SPR or Micro Scale Thermophoresis (MST) [35,36] and there was a correlation between the aminoglycoside riboswitch binding affinity and reporter gene expression. To investigate the interaction between the aminoglycosides and the *aad* riboswitch sequences, MST was used to measure aminoglycoside RNA binding. The riboswitch RNA *aad*-4 was prepared and labeled with fluorescein-5-thiosemicarbazide as described before [35]. The measurements were performed in a Monolith NT.115 system (NanoTemper Technologies) [43]. On titration of aminoglycosides increased binding was observed suggesting the formation of an aminoglycoside-RNA complex. The 4–6 deoxystreptamine aminoglycosides bound in the low µM range (from 1.2–3.9 µM), and display stoichiometric binding behavior with Hill constants close to one (Figure 3(c)), suggesting the formation of a 1:1 complex (Figure 3(a,b)). In comparison, the fragment antibiotics neamine (12 µM) and ribostamycin (40 µM) and the 4–5 deoxystreptamine paromomycin (10 µM) have much weaker affinities although their binding is also consistent with the formation of a 1:1 complex (Figure 3(b)). There was therefore a correlation between binding affinity and reporter gene expression, which is comparable to that for the published aac riboswitch such that the aminoglycosides that bind to *aad*-4 riboswitch RNA with higher affinities also induce reporter gene expression, and the control aminoglycosides that display lower affinities to the RNA do not induce reporter gene expression (Figure 3(c)).

**(a)** The 4–6 deoxystreptamine aminoglycoside Amikacin. **(b)** Binding curve generated by MST for binding of amikacin to the *aad-4* riboswitch. Error bars are standard deviations of at least three independent experiments. **(c)** Binding affinity of aad-4 riboswitch RNA with different aminoglycoside antibiotics measured by MST. Aminoglycoside dependent induction of gene expression for *aad-4* (Figure 2). Error bars are the standard deviations of at least three independent experiments.

### *Integron mediated site-specific recombination preserves the features and functions of the* aad *aminoglycoside riboswitches*

**Figure 4.**
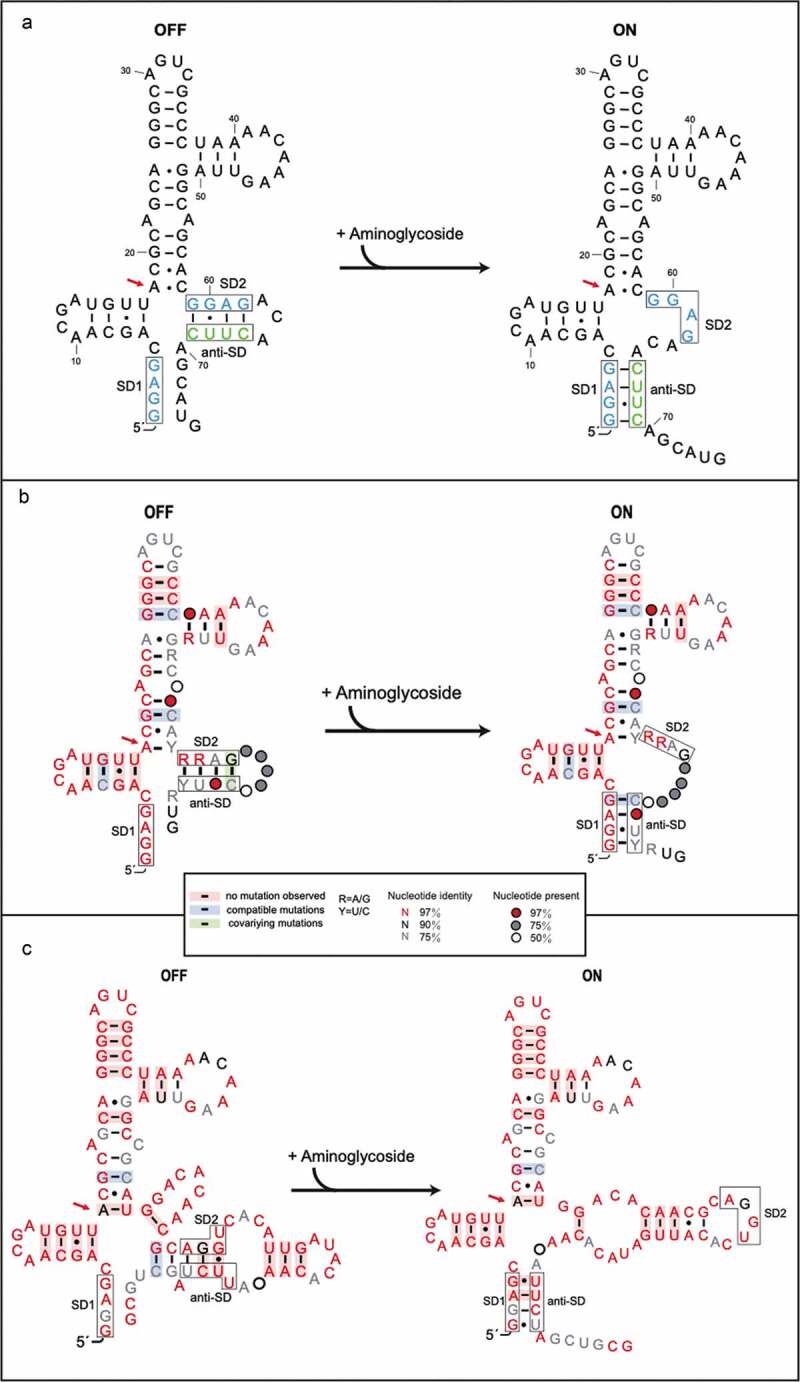
The structure transition model of *aac* riboswitch published and consensus model of *aac/aad* riboswitch in the presence or absence of aminoglycosides

A model was proposed for the regulatory mechanism of the *aac* riboswitches: in the absence of the antibiotic, the ribosome-binding site (SD2) of the riboswitch sequence was sequestered by a complementary anti-SD sequence, which blocked ribosome binding for translation; upon antibiotic binding a structural transition occurred leading to anti-SD base-paired with SD1 and free SD2 for ribosome binding/translation of the resistance gene [35,36,47] (Figure 4(a,b)). The *aad* riboswitch RNAs differ from the *aac* riboswitch RNA at the 3ʹend where the regulatory elements lie (Figure 1(b)). Despite the variation of regulatory elements in the *aad* riboswitch, they retain function as a riboswitch in the reporter assay. Further covariance analysis of the *aad* riboswitch RNA sequences revealed covariant structures and a potential structure transition (Figure 4(c)). We found that 29 bacterial strains including pathogenic strains such as *Pseudomonas aeruginosa*, and *Salmonella enterica* (Supplementary table 1) all share covariance structures and the potential for a structural transition. The first gene cassettes of the type 1 integrons can exploit lower frequency start codons and the *aad* genes are characterized by the presence of a low-frequency UUG initiation codon located 4–5 nt downstream of a consensus ribosome-binding site (SD2) sequence (AGGU) [11,15]. In the covariance analysis of the *aad* riboswitch sequences, although the SD2 (AGGU) and anti-SD2 (UUCU) sequence were variable, without the aminoglycosides SD2 is sequestered by the anti-SD2 sequence and, upon aminoglycoside binding, can potentially undergo a structural transition in which the anti-SD2 sequence pairs with SD1 and consequently makes SD2 available for ribosome binding. The sequences at SD2 and anti-SD2 of the riboswitch RNA are products of selective site-specific recombination by the integron so that bacteria survive in an antibiotic-rich environment through acquiring aminoglycoside resistance by activation of the *aad* gene. In the situation where recombination takes place in the absence of selection; the RNA sequences may fail to function as riboswitches and consequently inefficient translation of the *aad* RNAs may cause sensitivity to the antibiotic resulting in strain death. We note that the anti-SD2 sequence in the *aad* riboswitch is located immediately after the UUG initiation codon. In contrast, anti-SD2 sequence in the *aac* riboswitch is found before the AUG initiation codon. Nevertheless, in both cases integron-driven recombination selects viable riboswitch sequences and delineates essential features of the riboswitch.

**(a)** The secondary-structure of the *aac* riboswitch from *P. fluorescens* in the paper published by Jia et al [35]. The red arrow indicates the position of the inactive A18G point mutation. **(b)** The covariance model of *aac* riboswitch in the paper published by Wang et al ^(36)^. Red, black and gray nucleotides indicate the conservation of at least 97%, 90%, and 75%, respectively. The circles with red, gray, white color represent the positions in which nucleotide identity is less conserved as 97%, 75% and 50%, respectively. Base pairs shaded in red exhibit natural covariation, while those in blue exhibit compatible mutations. Purines and pyrimidines are identified by R and Y. The red arrow indicates the position of the inactive A18G point mutation. **(c)** The covariance model of the *aad* riboswitch RNA. Red, black and gray nucleotides indicate the conservation of at least 97%, 90%, and 75%, respectively. The circles with red, gray and white color represent the positions in which nucleotide identity is less conserved as 97%, 75% and 50%, respectively. Base pairs shaded in red exhibit natural covariation, while those in blue exhibit compatible mutations. Purines and pyrimidines are identified by R and Y. The red arrow indicates the position of the inactive A18G point mutation. The covariant structures were drawn using R2R and Adobe Illustrator.

### *The interaction between the* aad *riboswitch RNAs and the aminoglycosides are essential for the induction of gene expression*

Chemical probing data have previously shown that aminoglycoside binding to the *aac* riboswitch caused a structural transition such that the SD2 sequence became more accessible for translation. The A18 position of the aac riboswitch was identified as part of the aminoglycoside binding site by chemical probing and cross-linking experiments [35]. To investigate the importance of aminoglycoside interactions with the riboswitch and to test the proposed model shown in Figure 4, the point mutation A18G to the *aad* riboswitch was made and its activity with aminoglycosides examined by agar diffusion and Miller assays (Figure 2(b)). The single mutation A18G at the aminoglycoside binding site abolished induction of the reporter gene in the presence of aminoglycosides (Figure 2(b,g)) suggesting that the effect of the A18G mutation on the structured RNA was sufficient to prevent aminoglycoside dependent exposure of the SD2 sequence for translation initiation. Thus, the aminoglycoside dependent induction of gene expression requires interactions between the aminoglycoside and the riboswitch RNA that facilitates the structural transition that makes SD2 accessible.

There is a precedent for the translational regulation of ribosomal antibiotic-resistance gene expression through antibiotic-RNA-ribosome interactions. For the erythromycin resistance methytransferase, specific interactions between the amino acids of the nascent peptide and the peptide exit tunnel lead to ribosome stalling [20–22,48]. However, the A18G point mutation, located next to the initiation codon of the putative leader peptide, retains the amino acid sequence of the peptide and only changes the sequence of the RNA. Therefore, the loss of induction of the reporter gene in the A18G point mutant can only be due to the effect of the mutation on the RNA. Moreover, in the A18G mutant experiment, the aminoglycosides, the ribosome, and other possible factors of the translational machinery remain the same as in the wild type riboswitch. These results suggest that a specific interaction between the riboswitch RNA and aminoglycosides plays an important role in the translation of the reporter gene and support the proposed model in Figure 4(c).

## Discussion

Here the relationship between a class 1 integron (a site-specific DNA recombination system) at the DNA level and its associated aminoglycoside riboswitch in the 5ʹUTR of *aad* at the RNA level was investigated. The aminoglycoside-sensing riboswitch is linked with the class 1 integrons [35,36,45] and the DNA sequence of the 75-nucleotide aminoglycoside riboswitch RNA contains the recombinase binding site (*attI*). Blast searches of the aminoglycoside riboswitch identified new sequences from enduring aminoglycoside resistant pathogen strains, which are the end product of site-specific recombination by class 1 integrons. Analysis of the top six *aad* aminoglycoside riboswitch sequences revealed considerable sequence variation compared to that of *aac*; in particular the *aad* and *aac* riboswitches differ at the 3ʹend. Even single point mutations in the *aac* riboswitch can impair its function [35], but despite the variations in the *aad* sequences, the RNAs function as riboswitches in reporter assays (Miller assay and plate assays). Specific aminoglycoside-RNA binding was measured by MST (Figure 3(a,b), and Supplementary Figure-S2). Further covariance analysis of the *aad* riboswitch RNA sequences revealed potentially interchangeable covariant structures. The introduction of a single point mutation; A18G at a highly conserved position within each of the *aad* RNAs was sufficient to abolish aminoglycoside dependent gene expression. Specific aminoglycoside binding to the RNA may therefore induce the potential structural transition that unmasks SD2 for the initiation of translation. Taken together, these results indicate that at the DNA level, class 1 integrons carrying the *aad* gene cassettes underwent site-specific recombination to generate functional riboswitch RNAs that provide inducible aminoglycoside resistance. Active riboswitch RNAs were thus effectively selected for and retained by integron recombination because they conferred a selective advantage onto the recombinant product. The riboswitch provides an efficient mechanism that reduces the fitness burden on the cells such that the adenyltransferase genes are only expressed in the presence of the antibiotics [35]. The inducible aminoglycoside resistance riboswitches enable pathogenic strains to survive in an antibiotic-rich environment.

For the *aac* riboswitch RNA, each RNA sequence showed distinctive discrimination between different aminoglycosides in the reporter assay. For instance, among the aminoglycosides gentamicin-induced the highest levels of reporter gene expression for the *aac*-6 RNA, but sisomicin gave the highest reporter gene expression for *aac*-4 RNA and the optimal fold changes (~2-5 fold induction) were comparable to those observed in a clinical setting [36,49]. Here, in contrast to the *aac* riboswitch RNA, amikacin induces optimal reporter gene expression for each of the *aad* riboswitch RNAs that were investigated. The reporter assay allowed comparison of the *aac* and *aad* riboswitch RNAs in identical genetic backgrounds; the only difference in the assay is the different riboswitch RNAs. Both of the 5ʹUTR RNAs of the *aac* and *aad* genes have been preserved as functional riboswitches by the integron-mediated recombination.

The polycationic, flexible nature of the aminoglycosides might be expected to lead to nonspecific binding to polyanionic RNA. However, the differences in selectivity observed in both reporter assays and in equilibrium binding experiments [35,36] (Figure 2 Figure 3) suggest that the interactions between the aminoglycosides and the *aac* and *aad* riboswitch RNAs are probably due to specific interactions between the individual riboswitch RNA and the particular aminoglycoside. We have found that for each of the three RNAs in this and in previous studies [35,36], the 4,6 deoxystreptamine aminoglycosides bind to the RNA with higher affinity while 4,5 deoxystreptamine aminoglycosides bind to RNA weakly. Each RNA has an optimum binding pocket for a particular aminoglycoside. The specificity of aminoglycoside-RNA binding is conferred by interactions between the different RNAs and the functional groups on the drugs. Thus, each aminoglycoside confers different affinities and optimal induction for each different sequence.

Genetic recombination is an important evolutionary driving force that plays a key role in the generation of genetic diversity. In pathogenic bacteria the exposure to antibiotics has led to the specific acquisition of antibiotic-resistance genes through integron-mediated site-specific recombination. This flexible mechanism of gene transfer is an efficient survival response to the challenge of new and evolving classes of antibiotics. Aminoglycoside resistance emerged soon after their introduction to the clinic toward the middle of the last century [10,50]. The proliferation of antibiotic resistance through horizontal gene transfer is associated with the integron-mediated acquisition of antibiotic-resistance genes. Here we have shown that in clinical strains the capture of certain (adenyltransferases) genes by class 1 integrons confers a selective advantage to the integron through the creation of an aminoglycoside-sensing riboswitch.

## Supplementary Material

Supplemental Material
