# Supplementary material for: Aminoglycoside riboswitch control of the expression of integron associated aminoglycoside resistance adenyltransferases
Source: Virulence. 2020 Oct 24;11(1):1432–42. doi: 10.1080/21505594.2020.1836910 (PMC7588185; doi:10.1080/21505594.2020.1836910)
Supplement: Supplemental Material [file KVIR_A_1836910_SM5557.zip › Supplementary Figure Legends.docx]

**Supplementary Figure Legends**

**Supplementary Figure-S1:** The *β-gal* activity (Miller units) of the reporter gene with target riboswitch sequences upon titration of aminoglycosides. Target riboswitch sequences: (A) *aad*-1, (B) *aad*-2, (C) *aad*-3, (D) *aad*-5, (E) *aad*-6. Error bars are standard deviations of at least three independent experiments.

**Supplementary Figure-S2**

Agar diffusion assays of *E.coli* transformed with reporter construct plasmids of the aad and inactive aadA18G mutants in the presence of inducing and control aminoglycoside antibiotics (Kana B (kanamycin B), Genta (gentamicin), Amika (amikacin), Ribosta (ribostamycin), Paromo (paromomycin), Nea (neamine), Tobra (tobramycin) and Siso (sisomicin)) with IPTG; *aad*-1, *aad*-1 A18G, *aad*-3, *aad*-3 A18G, *aad*-5, *aad*-5 A18G, *aad*-6, *aad*-6 A18G are shown, in the assays expressions levels of *aad*-2 were too low for detection.

**Supplementary Figure-S3**: Binding curves generated by MST for binding of aminoglycoside antibiotics to the *aad*-4 leader RNA riboswitch. The aminoglycoside antibiotics are: (A) gentamicin, (B) kanamycin B, (C) sisomicin, (D) tobramycin, (E) paromomycin, (F) ribostamycin, (G) neamine. Error bars are standard deviations of at least three independent experiments.

**Supplementary Table 1**. The sequences that were used for the covariance model.

**Supplementary Table 2**. The sequences of *aad* (1-6).
