# Supplementary material for: Aminoglycoside riboswitch control of the expression of integron associated aminoglycoside resistance adenyltransferases
Source: Virulence. 2020 Oct 24;11(1):1432–42. doi: 10.1080/21505594.2020.1836910 (PMC7588185; doi:10.1080/21505594.2020.1836910)
Supplement: Supplemental Material [file KVIR_A_1836910_SM5557.zip › Supporting_Table_1.pdf]

Supplementary Table 1. The detail information of the sequences that were used for the covariance model.

Accession number	Organism	sequence
NG_052383.1/673-573	*Pseudomonas aeruginosa*	GUAGCAGCAACGAUGUUACGCAGCAGGGCAGUCGCCCUAAAACAAAGUUACGGCCGCAUGGACACAACGCAGGUCACAUUGAUACACCAAAUUCUACCUUCG
NG_052384.1/683-582	*Pseudomonas aeruginosa*	GGAGCAGCAACGAUGUUACGCACAGGGCAGUCGCCCUAAAACAAAGUUAGGCUUUUAUGGACACAACGCAGGUCACAUUGAUACACCAAAUUCUAGUUGCG
NG_052480.1/680-590	*Klebsiella pneumoniae*	GGAGCAGCAACGAUGUUUCGCAGCAGGGCAGUCGCCCUAAAACAAAGUUAAGCCGCAUGGACACAACGCAGGUCACAUUGAUACACAAAAUUCUAGCUGCG
NZ_CVRZ01000054.1/764-663	*Proteus vulgaris*	GGAGCAGCAACGAUGUUACGCAGCAGGGGCAGUCGCCCUAAAACAAAGUUAGGCCGCAUGGACACAACGCAGGUCACAUUGAUACACCAAAUUCUAGCUGCG
NZ_LXHA01000035.1/194-94	*Salmonella enterica*	GGAGCAGCAACGAUGUUACGCAGCAGGGCAGUCGCCCUAAAACAAGGGUAGGCCGCAUGGACACAACGCAGGUCACAUUGAUACACAAAAUUCGAGCGGCG
NZ_NBMT01000070.1/10604-10504	*Klebsiella pneumoniae*	GGAGCAGCAACGAUGUUACGCAGCAGGGCAGUCGCCCUAAAACAAAGU

		GAGGCCGCAUGGACACAACGCAGG UCACAUUGAUACACAAAAUUCUAG CUGCG
NZ_NFGJ01000069.1/100-200	*Pseudomonas aeruginosa*	GGAGCAGCAACGAUGUUACGCAGC AGGGCAGUCGCCCUAAAACAAAGU UAUGCCGCAUGGACACAACGCAGG UCACAUUGAUACACCAAAUUCUAG CUGCG
NC_009651.1/38256-38156	*Klebsiella pneumoniae*	GGAGCAGCAACGAUGUUACGCAGC AGGGCAGUCGCCCUAAAACAAGGU UAGGCCGCAUGGACACAACGCAGG UCACAUUGAUACACAAAAUUCUAG CUGCG
NZ_CXXE01000022.1/27-128	*Pseudomonas aeruginosa*	GGAGCAGCAACGAUGUUACGCAGC AGGGCAGUCGCCCUAAAACCAAAG UUAGGCCGCAUGGACACAACGCAG GUCACAUUGAUACACAAAAUUCUA GCUGCG
KU145267.1/70-170	*Acinetobacter baumannii*	GCAGCAGCAACGAUGUUACGCAGC AGGGCAGUCGCCCUAAAACAAAGU UAGGCCGCAUGGACACAACGCAGG UCACAUUGAUACACAAAAUUCUAG CUGCG
HM175864.1/16-117	*Pseudomonas aeruginosa*	GGAGCAGCAACGAUGUUACGCAGC AGGGCAGUCGCCCUAAAACAAAGU UAGGCCGCAUGGACACAACGCAGG UCACAUUGAUACACAAAAUUCUAG CUGCG
DQ343904.1/24-125	*Morganella morganii*	GCAGCAGCAACGAUGUUACGCAGC AGGGCAGUCGCCCUAAAACAAAGU UAGGCCGCAUGGACACAACGCAGG

		UCACAUUGAUACACAAAAUUCUAG CUGCG
DQ520939.1/15-116	*Proteus mirabilis*	GGAGCAGCAACGAUGUUACGCAGC AGGGCAGUCGCCCUAAAACAAAGG UUAGGCCGCAUGGACACAACGCAG GUCACAUUGAUACACAAAAUUCUA GCUGCG
EU746497.1/28-127	*Pseudomonas aeruginosa*	GGAGCAGCAACGAUGUUACGCAGC AGGGCAGUCGCCCUAAACAAAGUU AGGCCGCAUGGACACAACGCAGGU CACAUUGAUACACCAAAUUCUAGC UGCG
HM367617.1/1-102	*Pseudomonas aeruginosa*	GUAGCAGCAACGAUGUUACGCAGC AGGGCAGUCGCCCUAAAACAAAGU UACGGCCGCAUGGACACAACGCAG GUCACAUUGAUACACCAAAUUCUA CCUUCG
HQ880257.1/24-124	*Acinetobacter baumannii*	GGAGCAGCAACGAUGUUACGCAGC AGGGCAGUCGCCCUAAAACAAAGU UAGGCCGCAUGGACACAACGCAAG UCACAUUGAUACACAAAAUUCUAG CUGCG
JF714996.1/34-134	*Pseudomonas aeruginosa*	GGAGCAGCAACGAUGUUACGCAGC AGGGCAGUCGCCCUAAAACAAAGU UAGGCCGCAUGGACACAACGCAGG UCACAUUGAUACACCAAAUUCUAG CUGCG
DQ388125.1/6-106	*Salmonella enterica*	GGAGCAGCAACGAUGUUACGCAGC AGGGCAGUCGCCCUAAAACAAAGU UAGGCCGCAUGGACACAACGCAGG

		UCACAUUGAUACACAAAAUUCUAG<br>CUGCG
FJ908755.1/23-123	*Pseudomonas aeruginosa*	GGAGCAGCAACGAUGUUACGCAGC<br>AGGGCAGUCGCCCUAAAACAAAGU<br>UAGGCCGCAUGGACACAACGCAGG<br>UCACAUUGAUACACAAAAUUCUAG<br>CUGCG
HM367609.1/51-151	*Pseudomonas aeruginosa*	GGAGCAGCAACGAUGUUACGCAGC<br>AGGGCAGUCGCCCUAAAACAAAGU<br>UAGGCCGCAUGGACACAACGCAGG<br>UCACAUUGAUACACCAAAUUCUAG<br>CUGCG
AJ289190.2/1240-1343	*Acinetobacter baumannii*	GGAGCAGCAACGAUGUUACGCAGC<br>AGCAGGGCAGUCGCCCUAAAACAA<br>AGUUAGGCCGCAUGGACACAACGC<br>AGGUCACAUUGAUACACAAAAUUC<br>UAGCUGCG
AY139599.1/51-151	*Uncultured bacterium*	GGAGCAGCAACGAUGUUACGCAGC<br>AGGGCAGUCGCCCUAAAACAAAGU<br>UAGGCCGCAUGGACACAACGCAGG<br>UCGCAUUGAUACUCCAAUUUCUAG<br>CUGCG
KP420006.1/24-124	*Pseudomonas putida*	GGAGCAGCAACGAUGUUACGCAGC<br>AGGGCAGUCGCCCUAAAACAAAGU<br>UAGGCCGCAUGGACACAACGCAGG<br>UCACAUUGAUACACAAAAUUCUAG<br>CUGCG
AF458082.1/669-769	*Citrobacter freundii*	GGAGCAGCAACGAUGUUACGCAGC<br>AGGGCAGUCGCCCUAAACAAAGGU<br>UAGGCCGCAUGGACACAACGCAGG

		UCACAUUGAUACACAAAAUUCUAGCUGCG
EU247928.1/1052-948	*Klebsiella pneumoniae*	GGAGCAGCAACGAUGUUACGCAGCAGGCAGGGCAGUCGCCCUAAAACAAAGUUAGGCCGCAUGGACACAACGCAGGUCACAUUGAUACACAAAAUUCUAGCUGCG
HM367615.1/2-103	*Pseudomonas aeruginosa*	GGAGCAGCAACGAUGUUACGCACAGGGCAGUCGCCCUAAAACAAAGUUAGGCUUUUAUGGACACAACGCAGGUCACAUUGAUACACCAAAUUCUAGUUGCG
MF447896.1/9-110	*Klebsiella pneumoniae*	GGAGCAGCAACGAUGUUACGCAGCAGGGCAGUCGCCCUAAAACAAAGGUAGGCCGCAUGGACACAACGCAGGUCACAUUGAUACACAAAAUUCUAGCUGCG
AF364344.1/70-170	*Acinetobacter baumannii*	GCAGCAGCAACGAUGUUACGCAGCAGGGCAGUCGCCCUAAAACAAAGUUAGGCCGCAUGGACACAACGCAGGUCGCAUUGAUACUCCAAUUUCUAGCUGCG
X64368.1/283-382	*Klebsiella pneumoniae*	GGAGCAGCAACGAUGUUACGCAGCAGGCAGUCGCCCUAAAACAAAGUUAGGCCGCAUGGACACAACGCAGGUCACAUUGAUACACAAAAUUCUAGCUGCG
