# Supplementary material for: Aminoglycoside riboswitch control of the expression of integron associated aminoglycoside resistance adenyltransferases
Source: Virulence. 2020 Oct 24;11(1):1432–42. doi: 10.1080/21505594.2020.1836910 (PMC7588185; doi:10.1080/21505594.2020.1836910)
Supplement: Supplemental Material [file KVIR_A_1836910_SM5557.zip › Supporting_Table_2.pdf]

Supplementary Table 2. The detail information of sequences that were used for miller assay

**sequence label**	**sequence**
aad-1	GGAGCAGCAACGAUGUUACGCAGCAGGGCAGUCGCCCUAA<br>AACAAAGUUAGGCCGCAUGGACACAACGCAGGUCACAUUG<br>AUACACAAAAUUCUAGCUGCGGCAGAUGAGCGAAAU
aad-2	GCAGCAGCAACGAUGUUACGCAGCAGGGCAGUCGCCCUAA<br>AACAAAGUUAGGCCGCAUGGACACAACGCAGGUCGCAUUG<br>AUACUCCAAUUUCUAGCUGCGGCAGAUGAGCGAAAU
aad-3	GUAGCAGCAACGAUGUUACGCAGCAGGGCAGUCGCCCUAA<br>AACAAAGUUACGGCCGCAUGGACACAACGCAGGUCACAUU<br>GAUACACCAAAUUCUACCUUCGGCAGAUGAGCGAAAU
aad-4	GGAGCAGCAACGAUGUUACGCAGCAGGGCAGUCGCCCUAA<br>ACAAAGUUAGGCCGCAUGGACACAACGCAGGUCACAUUGA<br>UACACCAAAUUCUAGCUGCGGCAGAUGAGCGAAAU
aad-5	GGAGCAGCAACGAUGUUACGCAGCAGGGCAGUCGCCCUAA<br>AACAAAGUUAGGCCGCAUGGACACAACGCAAGUCACAUUG<br>AUACACAAAAUUCUAGCUGCGGCAGAUGAGCGAAAU
aad-6	GGAGCAGCAACGAUGUUACGCAGCAGGCAGUCGCCCUAAA<br>ACAAAGUUAGGCCGCAUGGACACAACGCAGGUCACAUUGA<br>UACACAAAAUUCUAGCUGCGGCAGAUGAGCGAAAU
