# Supplementary figures and images for: Aminoglycoside riboswitch control of the expression of integron associated aminoglycoside resistance adenyltransferases
Source: Virulence. 2020 Oct 24;11(1):1432–42. doi: 10.1080/21505594.2020.1836910 (PMC7588185; doi:10.1080/21505594.2020.1836910)

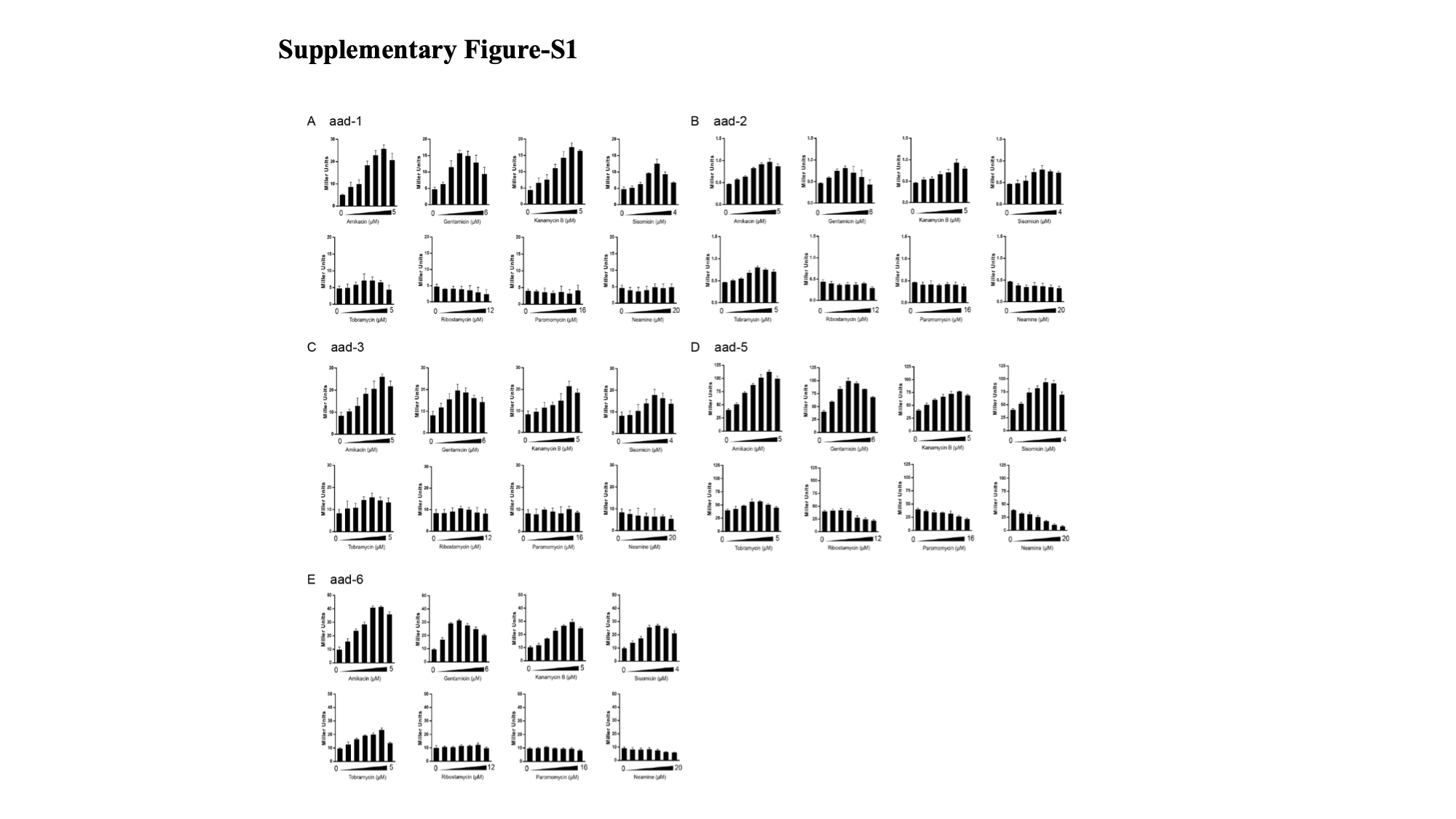

Supplement: Supplemental Material [file KVIR_A_1836910_SM5557.zip › Supplementary_Figure_1.tiff]

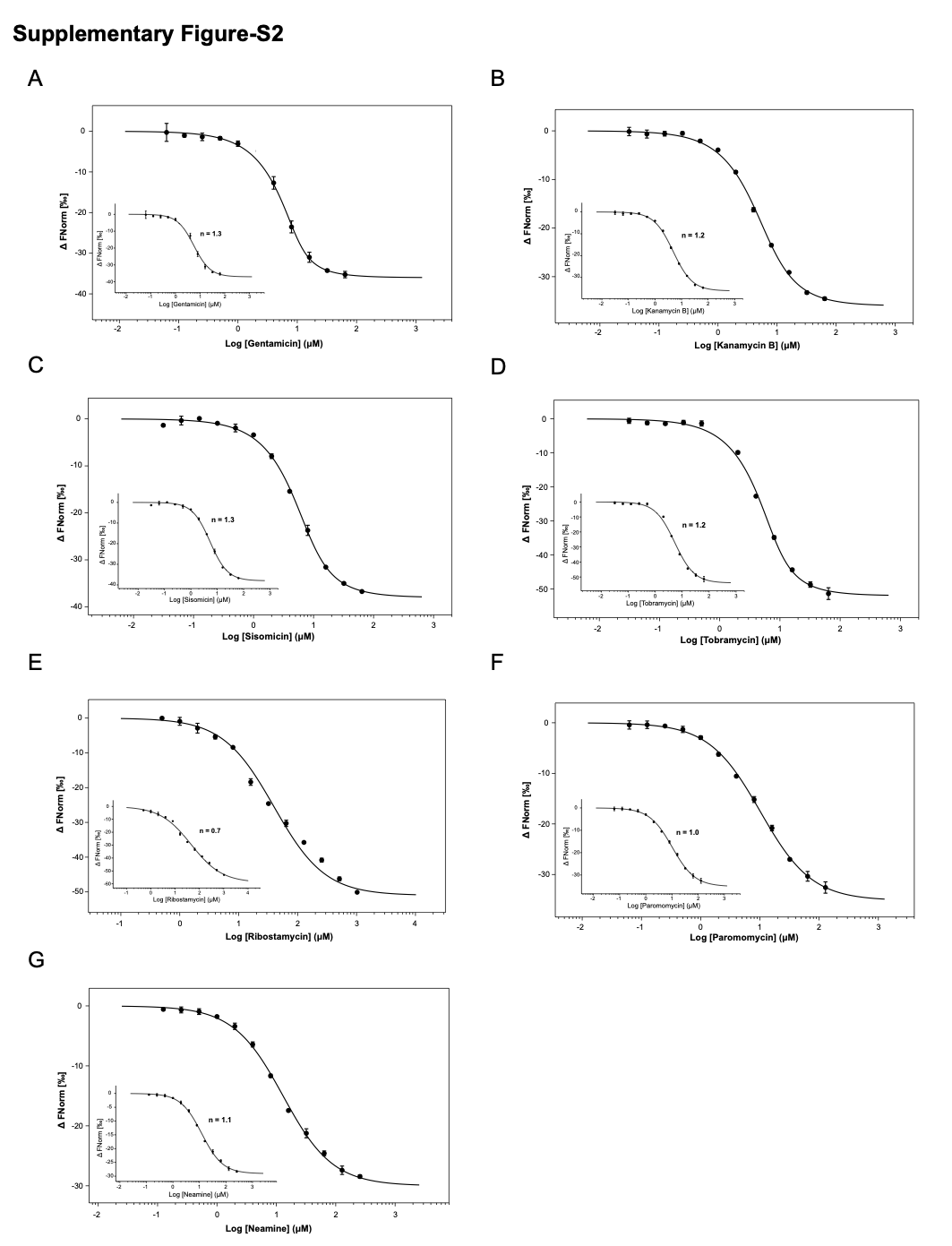

Supplement: Supplemental Material [file KVIR_A_1836910_SM5557.zip › Supporting_Figure3.tiff]

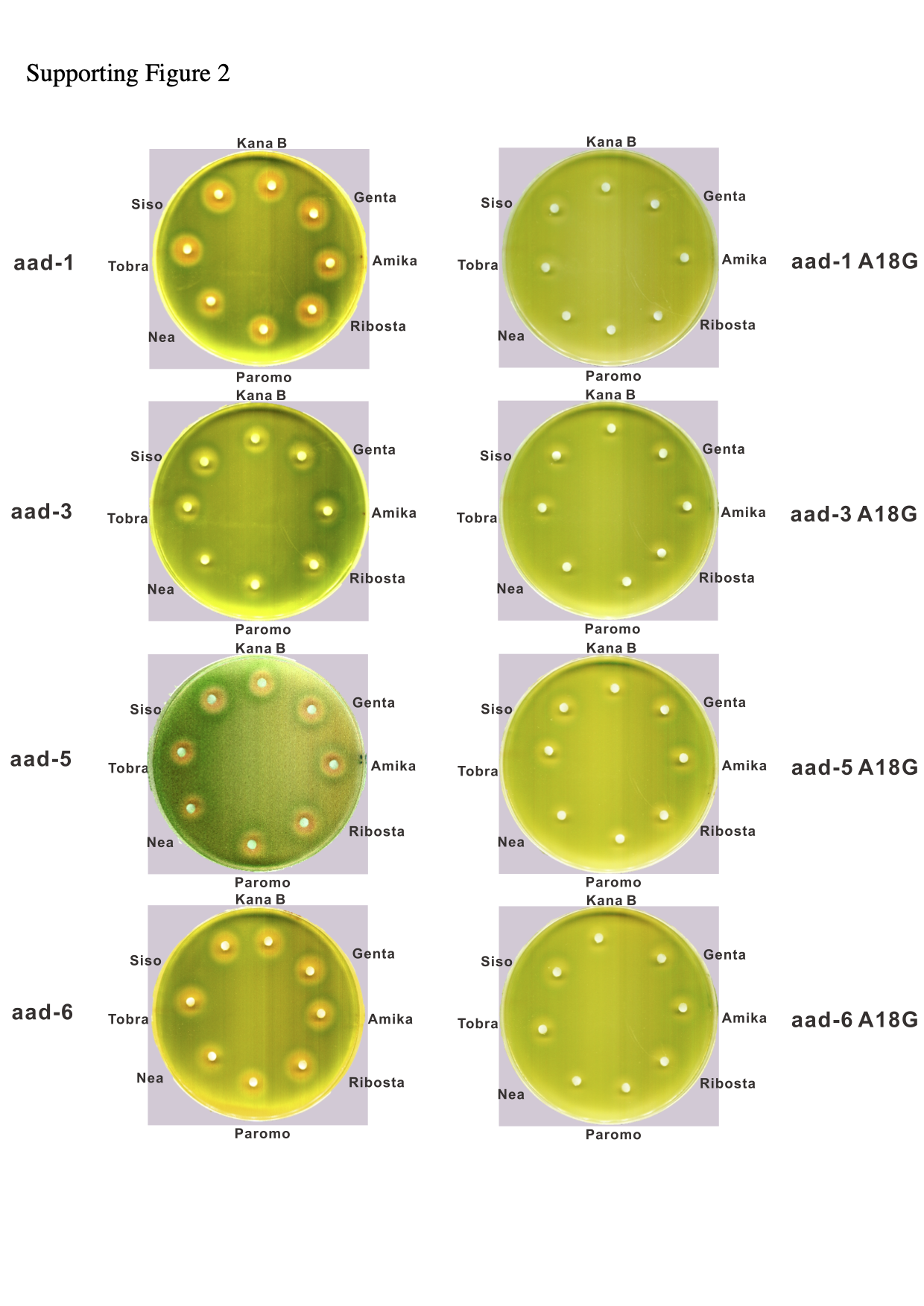

Supplement: Supplemental Material [file KVIR_A_1836910_SM5557.zip › Supporting_Figure_2.tiff]
